# Photoautotrophic Growth Rate Enhancement of *Synechocystis* sp. PCC6803 by Heterologous Production of 2-Oxoglutarate:Ferredoxin Oxidoreductase from *Chlorobaculum tepidum*

**DOI:** 10.3390/biology12010059

**Published:** 2022-12-29

**Authors:** June Kim, Eun Kyoung Oh, Eui-Jin Kim, Jeong K. Lee

**Affiliations:** 1 Department of Life Science, Sogang University, Seoul 121-742, Republic of Korea; 2 Microbial Research Department, Nakdonggang National Institute of Biological Resources, Sangju 37242, Republic of Korea

**Keywords:** 2-oxoglutarate:ferredoxin oxidoreductase, *Chlorobaculum tepidum*, reductive TCA cycle, carboxylation, photoautotrophic growth, *Synechocystis*

## Abstract

**Simple Summary:**

*Synechocystis* sp. PCC6803 (*Synechocystis*) is a photosynthetic organism useful for biotechnological applications. It utilizes light energy and fixes CO_2_ to synthesize C3 organic acids for growth and biomass formation. However, carbon fixation is the limiting step for its maximal growth. To enhance the carbon fixation, 2-oxoglutarate:ferredoxin oxidoreductase from *Chlorobaculum tepidum* (CtOGOR)—a carbon-fixing enzyme in the reductive TCA cycle—was produced *in trans* in *Synechocystis*. Overexpression of the CtOGOR gene effectively altered 2-oxoglutarate and glutamate levels and elevated the photoautotrophic growth rate of *Synechocystis*.

**Abstract:**

2-Oxoglutarate:ferredoxin oxidoreductase from *Chlorobaculum tepidum* (CtOGOR) is a carbon-fixing enzyme in the reductive TCA cycle that reversibly carboxylates succinyl-CoA to yield 2-oxoglutarate. CtOGOR is a heterotetramer of two large (α = 68 kDa) and two small (β = 38 kDa) subunits. The αβ protomer harbors one thiamine pyrophosphate and two 4Fe-4S clusters. Nonetheless, the enzyme has a considerable oxygen tolerance with a half-life of 143 min at 215 μM dissolved oxygen. Kinetic analyses of the purified recombinant CtOGOR revealed a lower *K*_m_ for succinyl-CoA than for 2-oxoglutarate. Cellular levels of 2-oxoglutarate and glutamate—a product of glutamine oxoglutarate aminotransferase and glutamate dehydrogenase—increased more than twofold in the exponential phase compared with the control strain, leading to an approximately >30% increase in the photoautotrophic growth rate. Thus, CtOGOR was successfully produced in *Synechocystis*, thereby boosting carboxylation, resulting in enhanced photoautotrophic growth.

## 1. Introduction

**Figure 1:**
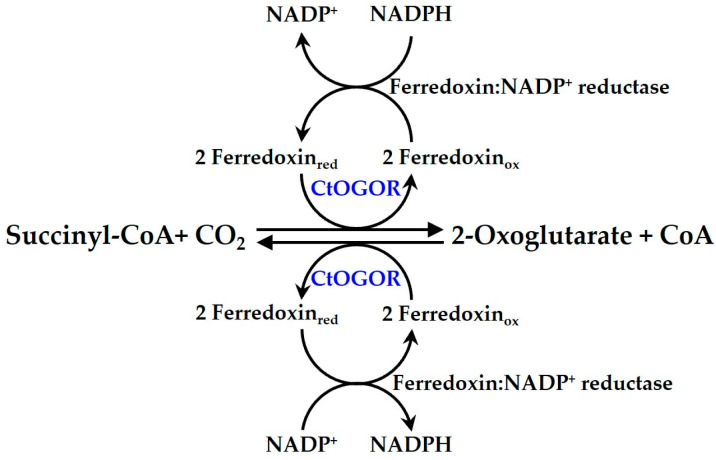
The reaction scheme of CtOGOR. The enzymatic reaction of CtOGOR is illustrated including a coupled reaction with ferredoxin:NADP^+^ reductase (FNR) for in vitro assays.

*Chorobaculum tepidum* is a photosynthetic bacterium that possesses a reductive TCA (rTCA) cycle for carbon fixation [1,2]. Among the carbon-fixing enzymes responsible for critical steps of the rTCA cycle, it possesses 2-oxoglutarate:ferredoxin oxidoreductase (OGOR, also known as KGOR or KFOR), which is classified under the 2-oxoacid:ferredoxin oxidoreductase (OFOR) superfamily. It catalyzes the bidirectional carboxylation/decarboxylation reaction between succinyl-CoA and 2-oxoglutarate (Figure 1). OGORs from diverse species have been previously studied [3,4,5,6,7,8]. OGORs commonly contain the [4Fe-4S] cluster and thiamine pyrophosphate (TPP), although the number of each cofactor varies [3,4,5,6,7,8]. Due to the (4Fe-4S) cluster, the OFOR superfamily of enzymes is generally vulnerable to oxygen exposure [9]. OGOR from *C. tepidum* (CtOGOR) consists of a large subunit (CtOGOR-A, α-subunit, KorA) and small subunit (CtOGOR-B, β-subunit, KorB) [1].

*Synechocystis* sp. PCC6803 (hereafter *Synechocystis*) is a phototrophic cyanobacterium that depends on ribulose-1,5-bisphosphate carboxylase-oxygenase (RuBisCO) for carbon fixation. Recently, *Synechocystis* has attracted considerable attention for its light-utilizing biotechnological applications [10]. Similar to plants and other phototrophic organisms, carbon fixation is a rate-limiting step for the effective production of *Synechocystis* biomass because of the low turnover rate (approximately 3.3 s^−1^ in spinach, 9–14 s^−1^ in *Synechocystis*) and low substrate specificity of RuBisCO [10,11,12,13,14]. Accordingly, several studies have been conducted to increase the carbon fixation rate by overexpressing RuBisCO and other enzymes in the Calvin-Benson-Bassham (CBB) cycle to increase the yield of biomass and carbonaceous product [15,16,17].

Here, we biochemically characterized CtOGOR and heterologously produced it in *Synechocystis* to promote carbon fixation. The product of CtOGOR is 2-oxoglutarate, which is readily (trans)aminated to glutamate by either glutamine oxoglutarate aminotransferase (GOGAT) or glutamate dehydrogenase (GDH). Considering the metabolic flux levels leading to biomass, glutamate is known to greatly enhance the biomass yield of *Synechocystis* [18].

## 2. Materials and Methods

### 2.1. Bacterial Strains and Growth Conditions

For photoautotrophic growth, *Synechocystis* was cultured in BG-11 minimal medium [19] at 30 °C. The culture was agitated by a shaking incubator at 130 rpm in 250-mL Erlenmeyer flasks under fluorescent light of 40 μmole·m^−2^·s^−1^. *Escherichia coli* was cultured at 37 °C in Luria-Bertani (LB) medium [20] with agitation at 250 rpm. Antibiotics were added to the medium when necessary, at the following concentrations: For *Synechocystis*, gentamicin (Gm) was added at 30 μg/mL; For *E. coli*, ampicillin (Ap) and Gm were added at 50 and 30 μg/mL, respectively.

### 2.2. Construction of Plasmids and Their Mobilization into Synechocystis

All plasmids were constructed in *E. coli* DH5α*phe* [21]. *korA* (CT0163) and *korB* (CT0162), which encode the α and β subunits of CtOGOR, respectively, are clustered around a 3-bp gap in the genome of *C. tepidum*. The open reading frames (ORFs) of the genes were synthesized using optimized codons for *E. coli*. The synthesized *korA* fragment was flanked by *Xho*I and *Hin*dIII sites. The *korB* fragment was flanked by *Hin*dIII and *Eco*RI sites with a strep-tag sequence (5′-TGGAG CCACC CGCAG TTCGA AAAA-3′) between the penultimate and stop codons. Both fragments were cloned into *Xho*I and *Eco*RI sites of pRSET-A (Invitrogen, Waltham, MA, USA) to form pRSET-ogor, and N-terminal His_6_-tagged CtOGOR-A and C-terminal strep-tagged CtOGOR-B were produced in *E. coli*. In the pRSET-ogor, the gap between *korA* and *korB* was 6-bp and the ribosomal binding site of *korB* in *korA* (the region including the last 20 bp sequence of *korA* ORF) was the same as the original genomic DNA sequence.

The *korAB* fragment in pRSET-ogor was PCR-amplified using the primer set 5′-AAAAA ACTCG AGGAA GGAGA TATAC AAATG AGTGA TACCG TAATC-3′ and 5′-AAAAA AGAAT TCTTA ATTAA TGGTC CACGT GCT-3′, followed by digestion with *Xho*I and *Eco*RI and cloned into the same sites of pSL1211 [22], resulting in pSL-ogor. pSL-ogor produced CtOGOR which is free of His_6_- or strep-tags, following isopropyl β-D-1-thiogalactopyranoside (IPTG) induction in *Synechocystis*. pSL-ogor was mobilized into *Synechocystis* via conjugation with *E. coli* S17-1 [23] carrying pSL-ogor, as described previously [24].

To purify ferredoxin-NADP^+^ reductase (FNR) and ferredoxin (Fd) of *Synechocystis* from *E. coli*, the ORF of FNR (slr1643) was PCR-amplified from the genomic DNA of *Synechocystis* using the primer set 5′-AAAAA AGGAT CCATG TACAG TCCCG GTTAC-3′ and 5′-AAAAA AAAGC TTTTA GTAGG TTTCC ACGTG-3′, and that of Fd (slr1382) was amplified with the primer set 5′-AAAAA AGGAT CCATG TCCCG TTCCC ACCGA-3′ and 5′-AAAAA AAAGC TTCTA GTCCT CATCT AAAGG C-3′. FNR and Fd gene fragments were digested with *Bam*HI/*Hin*dIII and ligated into the same sites of pQE30 (Qiagen, Hilden, Germany), resulting in pQE-fnr and pQE-fd, respectively.

To purify *Synechocystis* isocitrate dehydrogenase (Icd) in *E. coli*, ORF of Icd (slr1289) was PCR-amplified from the genomic DNA of *Synechocystis* using the primer set 5′-AAAAA AGGAT CCATG TACGA AAAAC TTCAG-3′ and 5′-AAAAA AAAGC TTTTA ATCAT CGAAA TGACT-3′, followed by digestion with *Bam*HI/*Hin*dIII and subsequent ligation into the same sites of pRSET-A, resulting in pRSET-icd.

To purify aminolevulinic acid synthase (ALAS) of *Rhodobacter sphaeroides* 2.4.1, the ORF (RSP_2984) without a stop codon was amplified from the genomic DNA of *R. sphaeroides* 2.4.1 using the primer set 5′-AAAAA ACATA TGGAC TACAA TCTGG CACT-3′ and 5′-AAAAA AAAGC TTGGC AACGA CCTCG GCGCG AT-3’. The fragment was digested with *Nde*I/*Hin*dIII and ligated into the same sites of pET29a (Merck, Darmstadt, Germany), resulting in pET-alas.

### 2.3. Purification of Recombinant Enzymes from E. coli

To purify CtOGOR, *E. coli* BL21 (DE3) (Stratagene, San Diego, CA, USA) transformed with pRSET-ogor was cultivated at 37 °C and agitated at 250 rpm to an OD_600_ of 0.6. IPTG (1 mM) was added to the culture medium, followed by incubation at 30 °C for 18 h with agitation at 250 rpm. All the following procedures were conducted in an anaerobic chamber (Model 10; Coy Laboratory Products, Grass Lake, MI, USA) filled with a gas mix of 90% N_2_/5% H_2_/5% CO_2_. The cells were harvested by centrifugation at 6000× *g* at 4 °C for 10 min and resuspended in Buffer-A (50 mM Na_2_HPO_4_ (pH 8.0), 300 mM NaCl, 10 mM imidazole). The cells were lysed by sonication (5 min lysis/5 min rest, 3 cycles, on ice), followed by centrifugation at 8000× *g* at 4 °C for 10 min to obtain the lysate. The lysate was loaded onto Ni-NTA agarose resin (Qiagen) and washed with five resin volumes of Buffer-A (His-tag affinity purification). The enzyme was eluted using Buffer-A supplemented with 240 mM imidazole. The purity of the CtOGOR in the eluate was >99%.

For further purification and assessment of the native molecular mass, the eluted CtOGOR was subjected to gel-filtration chromatography. The sample was loaded into Superdex^TM^ 200 prep-grade resin (Cytiva, Emeryville, CA, USA) with dimensions of 10 mm/300 mm (diameter/height). The column was eluted with 50 mM Na_2_HPO_4_ (pH 8.0) containing 300 mM NaCl at a flow rate of 0.25 mL/min, and the absorbance at 280 nm was recorded. Thyroglobulin (669 kDa; cat. #. T1001; Sigma-Aldrich, St. Louis, MO, USA), ferritin (480 kDa; cat. #. F4503; Sigma-Aldrich), catalase (240 kDa; cat. #. C9322; Sigma-Aldrich), albumin (67 kDa; cat. #. A2153; Sigma-Aldrich), and cytochrome *c* (12.3 kDa; cat. #. C7150; Sigma-Aldrich) were used as size standards. Western blotting of purified CtOGOR with antibodies against His_6_- and strep-tags was performed as described previously [25]. *E. coli* BL21 (DE3) transformed with pQE-fnr, pQE-fd, pRSET-icd and pET-alas was used for the purification of FNR, Fd, Icd, and ALAS, respectively, using His-tag affinity purification.

### 2.4. Reconstitution and Determination of Cofactors in CtOGOR

The iron-sulfur clusters of CtOGOR were reconstituted using cysteine desulfurase (IscS), as previously described [25,26]. After reconstitution of the iron-sulfur cluster, TPP was added at a 10-fold concentration of CtOGOR and incubated at 4 °C for 1 h. The sample was dialyzed against 50 mM Na_2_HPO_4_ (pH 7.5) at 4 °C for 24 h. The iron [27], labile sulfur [28], and TPP [29] contents were determined as previously described. The amount of CtOGOR was quantified using the Lowry assay [30].

### 2.5. Kinetic Analyses of Enzymes

All reactions were performed under anaerobic conditions at 30 °C. The basal mix contained 50 mM Na_2_HPO_4_ (pH 7.5), 1 mM MgCl_2_, 1 mM DTT, 1 mM TPP, 0.1 μM FNR, 1 μM Fd, and 0.2 μM CtOGOR (reconstituted with iron-sulfur cluster and TPP). In the carboxylation reaction, 0.01–0.1 mM succinyl-CoA with 50 mM sodium bicarbonate (fixed), or 5–50 mM sodium bicarbonate with 0.5 mM succinyl-CoA (fixed) were added to the basal mix. The reactions were initiated by adding 0.15 mM NADPH, and the decrease in A_340_ was recorded. In the decarboxylation reaction, 0.1–10 mM disodium 2-oxoglutarate with 0.5 mM CoA (fixed), or 0.01–0.1 mM CoA with 5 mM disodium 2-oxoglutarate (fixed) were added to the basal mix. The reactions were initiated by adding 0.15 mM NADP^+^ and the increase in A_340_ was recorded. The initial velocities were calculated from the changes in A_340_. The datasets were fitted to Michaelis-Menten equation to determine the kinetic parameters using SigmaPlot ver. 14 (Systat Software, San Jose, CA, USA). Initial concentrations of CO_2_ were estimated from those of sodium bicarbonate by using p*K*_a_ of 6.11 in equilibrium between CO_2_ and HCO_3_^−^ [31,32].

The kinetic analysis of Icd was performed in 10 mM HEPES (pH 8.0) containing 40 mM MgCl_2_ and 2 nM Icd. In the carboxylation reaction, 0.2–1.6 mM disodium 2-oxoglutarate with 50 mM sodium bicarbonate (fixed), or 5–40 mM sodium bicarbonate with 5 mM disodium 2-oxoglutarate (fixed) were added, which were initiated by adding 0.15 mM NADPH. In the decarboxylation reaction, 0.1–0.8 mM trisodium isocitrate and 0.15 mM NADP^+^ were added. The initial velocities were calculated from the changes in the A_340_. All analyses were performed as described for CtOGOR. Initial concentrations of CO_2_ were estimated from those of sodium bicarbonate using a p*K*_a_ of 6.11 in equilibrium between CO_2_ and HCO_3_^−^ [31,32].

### 2.6. Assessment of O_2_ Stability of CtOGOR

The 50 mM Na_2_HPO_4_ (pH 7.5) buffer was pre-equilibrated with gas mixtures containing varying levels of O_2_, as previously described [33]. The purified CtOGOR was incubated in the buffers and aliquots were taken intermittently to determine the residual carboxylation activity, as mentioned earlier.

### 2.7. Total RNA Extraction and Quantitative Reverse Transcription PCR (RT-qPCR)

The procedures and reagents for total RNA extraction and RT qPCR were essentially the same as those previously described [25]. The transcript level of *korB* was determined using the primer set 5′-GGAGC TTTTA GAGCC TTCTC-3′ and 5′-CTCTT TTCGA GGTCA ATCAC-3′. The expression of *rbcL* (slr0012) was determined using the primer set 5′-TTGGA CTGAC AACCT AACTG-3′ and 5′-ATACG TTACC GACCA AAGAG-3′. The 16S rRNA gene (*rrn16Sa*) was used as a reference gene and was amplified by the primer set 5′-TACAG TAGGG GTAGC AGGAA-3′ and 5′-GGCTA GGACT ACAGG GGTAT-3′. Relative changes in transcript levels were assessed using the comparative C^T^ method [34].

### 2.8. Preparation and Spectral Analysis of the Membrane Fraction

Cells harvested during the exponential phase were resuspended in 10 mM phosphate-buffered saline (PBS) at pH 7.4 and lysed by sonication. After centrifugation at 6000× *g* and 4 °C for 10 min, the supernatant was decanted to perform ultracentrifugation at 150,000× *g* and 4 °C for 1 h. Then, the resulting pellet was resuspended in PBS supplemented with 1% (*w*/*v*) n-dodecyl β-D-maltoside and mixed by inverting at 4 °C for 1 h. Insoluble materials were removed by centrifugation at 12,000× *g* and 4 °C for 5 min. The supernatant (membrane fraction) was subjected to absorption spectrum measurements using a UV-Vis spectrophotometer (UV 2550; Shimadzu, Kyoto, Japan).

### 2.9. Determination of Cellular Enzyme Activity

The cellular carboxylation activity of OGOR was determined as described above in the kinetic analyses of the enzyme sections, which were also performed under anaerobic conditions. *Synechocystis* cells were harvested during the exponential phase, resuspended in 50 mM Na_2_HPO_4_ (pH 7.5), and lysed by sonication, as described earlier for enzyme purification. The reaction was performed with cell lysate (0.1 mg protein) in the basal mix, from which CtOGOR was omitted and further supplemented with 0.15 mM NADPH and 50 mM NaHCO_3_. The reaction was initiated by adding 0.5 mM succinyl-CoA. The background control was conducted in the absence of succinyl-CoA. Cellular RuBisCO activity was determined as described previously using *Synechocystis* lysates [35].

### 2.10. Determination of Metabolite Levels

The cells were harvested during the exponential phase and resuspended in 50 mM Tris-Cl (pH 8.0). The cells were lysed by sonication and unbroken cells were removed by centrifugation at 12,000× *g* for 5 min. The protein contents in the supernatant were determined using the Lowry assay. The supernatants were filtered through a membrane with a 3-kDa cutoff (Amicon^TM^ Ultra; Merck) to remove cellular proteins. The filtrates were used for metabolite determination. Succinyl-CoA was determined by a coupled assay using ALAS. The filtrates were supplemented with 0.5 mM PLP, 50 mM glycine, and 5 μM ALAS, and incubated at 30 °C for 15 min. The reaction without ALAS was included as a background control. After the reaction, the succinyl-CoA in the samples was completely converted to 5-aminolevulinic acid which was quantified as previously described [36]. The levels of 2-oxoglutarate (cat. #. K677-100; BioVision, Milpitas, CA, USA), glutamate (cat. #. K629-100; BioVision), succinate (cat. #. K649-100; BioVision) and isocitrate (cat. #. MAK061; Sigma-Aldrich) were determined using the commercial assay kits.

## 3. Results

### 3.1. Biochemical Characterization of CtOGOR

**Figure 2:**
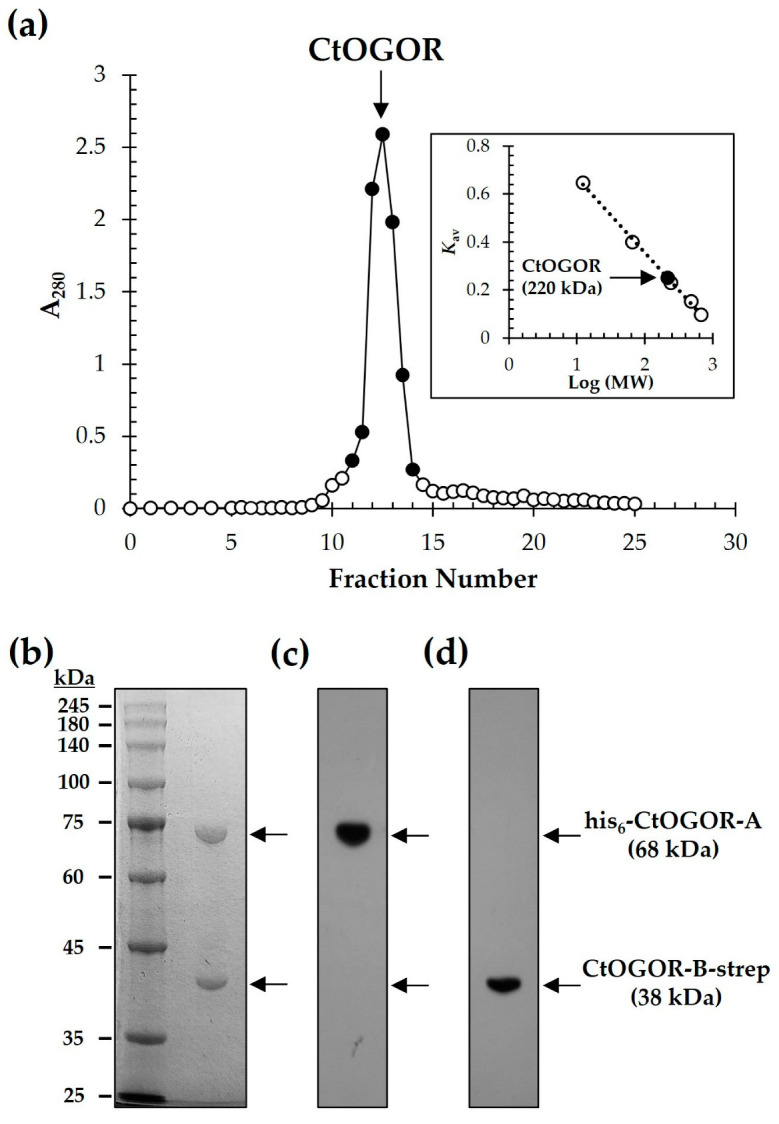
Purification of 2-oxoglutarate:ferredoxin oxidoreductase from *Chlorobaculum tepidum* (CtOGOR). CtOGOR composed of N-terminal His_6_-tagged CtOGOR-A (His_6_-CtOGOR-A) and C-terminal strep-tagged CtOGOR-B (CtOGOR-B-strep) was purified by His-tag affinity chromatography, followed by gel-filtration chromatography using Sephacryl^TM^ S-300 HR (**a**). The molecular mass of native CtOGOR was determined to be 220 kDa, which was calculated by the standard curve constructed with size standards (inset of A). Fractions of major peak (closed circle) were pooled and separated by SDS-PAGE (**b**). The His_6_-CtOGOR-A and CtOGOR-B-strep were confirmed by Western blotting using antibody against His_6_-tag (**c**) and strep-tag (**d**), respectively.

CtOGOR is composed of two subunits, CtOGOR-A (α-subunit) and CtOGOR-B (β-subunit). The ORFs of *korA* and *korB*, coding for CtOGOR-A and CtOGOR-B, respectively are clustered around a 3-bp gap in the genome of *C. tepidum*, which implies that both genes are co-expressed at the transcriptional level as well as the tight interaction of gene products. As expected, after purification using His-tag affinity chromatography and subsequent gel-filtration chromatography, a single peak was detected in the chromatogram, which corresponded to a molecular mass of 220 kDa (Figure 2a). When the peak was pooled and subjected to SDS-PAGE, two bands were observed (Figure 2b and Figure S1a), illustrating the expected size of both subunits of CtOGOR at similar densities. These were confirmed to be His_6_-CtOGOR-A (N-terminally His_6_-tagged CtOGOR-A) and CtOGOR-B-strep (C-terminally strep-tagged CtOGOR-B) by Western blotting experiments

(Figure 2c,d and Figure S1b,c). This indicated that the two subunits were co-expressed and tightly bound to each other. Assuming that CtOGOR-A and CtOGOR-B constitute a multimer in 1:1 molar ratio, the oligomeric state of native CtOGOR is suggested to be the heterotetrameric (αβ)_2_ form.

The cofactor content of CtOGOR was determined after reconstitution of the enzyme with iron, sulfur, and TPP. CtOGOR was found to contain 7.51 ± 0.51, 7.65 ± 0.12, and 0.77 ± 0.07 of Fe, S, and TPP, respectively, per αβ-protomer (Table 1). If the iron-sulfur cluster is (4Fe-4S)-type like other OFOR superfamily enzymes [3,4,5,6,7,8], the CtOGOR protomer has two (4Fe-4S) clusters and one TPP as its cofactor.

Because the direction of the CtOGOR reaction is reversible (Figure 1), kinetic parameters were determined for both carboxylation and decarboxylation (Table 2). Enzyme analysis was performed with *Synechocystis* Fd as a mediator for electron transfer at 30 °C to elucidate the effect of CtOGOR production on the photoautotrophic growth of *Synechocystis*. The *K*_m_ values varied among the substrates. However, there was a notably large difference found between *K*_m_ for the major carbon substrates, succinyl-CoA (C_4_ compound) and 2-oxoglutarate (C_5_ compound). The *K*_m_ for succinyl-CoA was 71-fold lower than that for 2-oxoglutarate, indicating that the affinity of succinyl-CoA for CtOGOR was far higher than that of 2-oxoglutarate (Table 2). If the concentrations of CO_2_ and CoA are sufficiently high and those of succinyl-CoA and 2-oxoglutarate are comparable, the reaction would move toward the carboxylation direction. This expectation appears to be obvious from the 23-fold higher catalytic efficiency (*k*_cat_/*K*_m_) for succinyl-CoA than that for 2-oxoglutarate.

**Figure 3:**
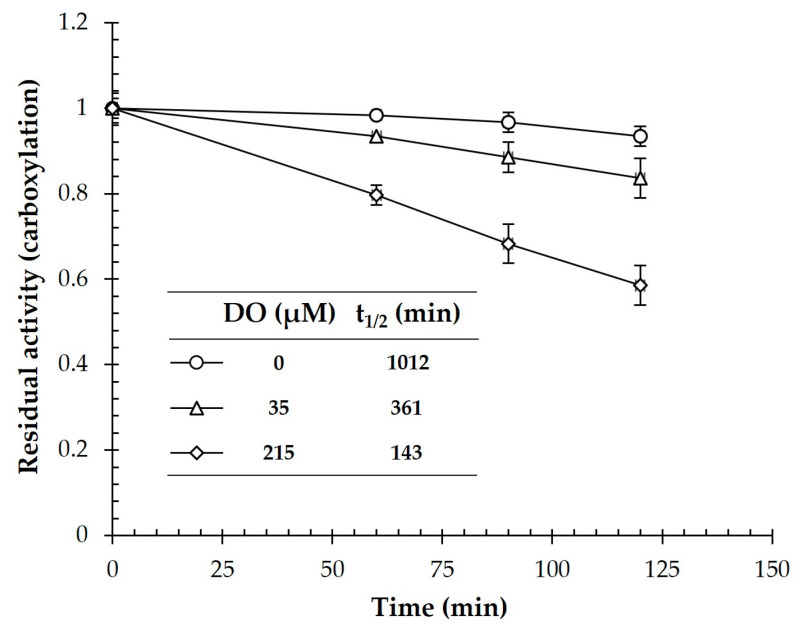
Stability of 2-oxoglutarate:ferredoxin oxidoreductase (CtOGOR) at varying O_2_ levels. CtOGOR was incubated with the buffers equilibrated with gases containing varying levels of O_2_, and the residual carboxylation activities were recorded intermittently. The half-lives (t_1/2_) were extrapolated by trendlines.

*Synechocystis* performs oxygenic photosynthesis. Thus, molecular oxygen is produced by the light reaction of photosynthesis, and the Fe-S cluster of CtOGOR may be detrimentally affected. Enzymes in the OFOR superfamily are sensitive to O_2_ [9]. Accordingly, we assessed the stability of the carboxylation activity of CtOGOR under anaerobic (0% O_2_: DO, 0 μM), semi-aerobic (2% O_2_: DO, 35 ± 5 μM), and aerobic (21% O_2_: DO, 215 ± 7 μM) conditions (Figure 3). The residual activity decreased further as the DO level increased. Nonetheless, CtOGOR maintained its activity with a half-life of 143 min under aerobic conditions. Moreover, the half-life was extended to 361 min under semi-aerobic conditions, which may correspond to the O_2_ environments in *Synechocystis* cells [24].

### 3.2. Heterologous Production of CtOGOR Enhanced Photoautotrophic Growth Rate of Synechocystis by Approximately >30%

**Figure 4:**
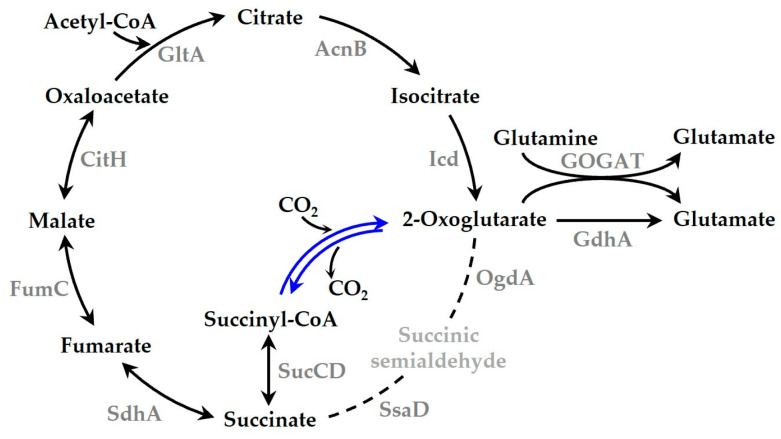
Tricarboxylic acid (TCA) cycle of *Synechocystis* producing 2-oxoglutarate:ferredoxin oxidoreductase (CtOGOR) under photoautotrophic conditions. CtOGOR (blue lines) is produced in *Synechocystis* and the TCA cycle is illustrated with its metabolites and enzymes. The dotted lines are supposed to be least active in the photoautotrophic condition [38]. Several cofactors necessary for enzyme reactions such as ATP and NAD(P)H are omitted. Abbreviations: GltA, citrate synthase; AcnB, aconitate hydratase B; Icd, isocitrate dehydrogenase; GOGAT, Glutamine oxoglutarate aminotransferase; GdhA, glutamate dehydrogenase; OgdA, 2-oxoglutarate decarboxylase; SsaD, succinic semialdehyde dehydrogenase; SucCD, succinyl-CoA synthetase; SdhA, succinate dehydrogenase subunit A; FumC, fumarase C; CitH, malate dehydrogenase [39].

**Figure 5:**
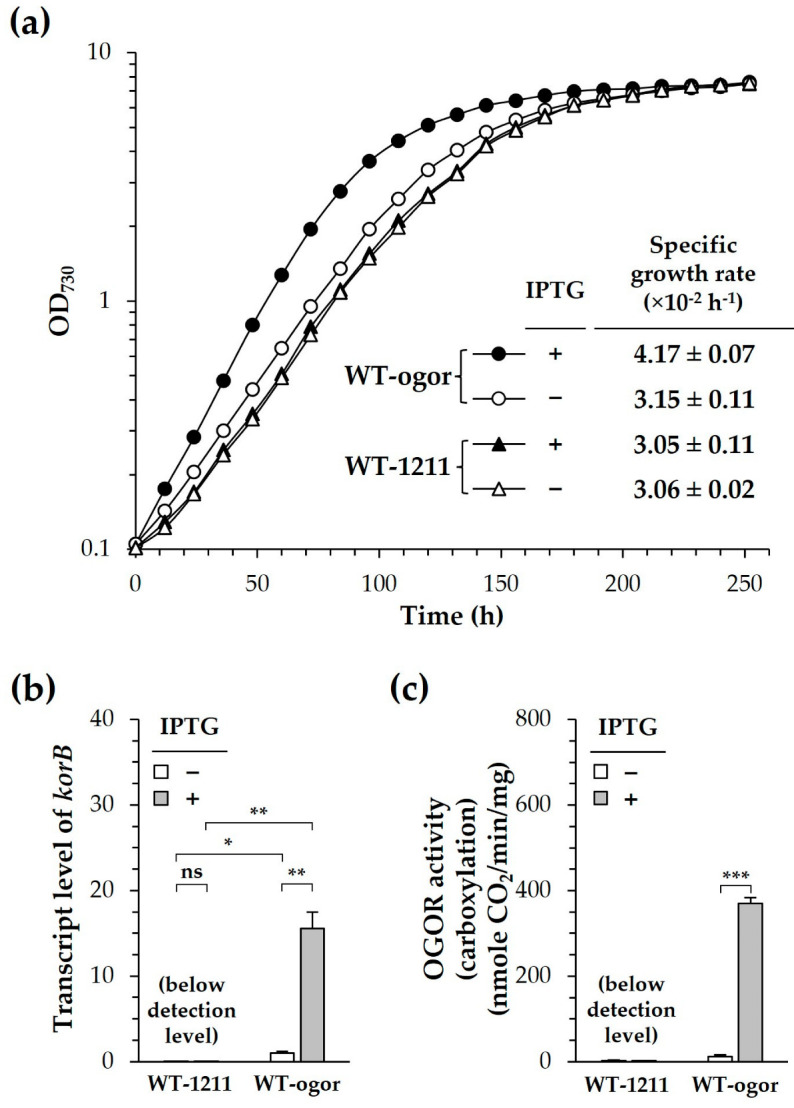
Photoautotrophic growth of *Synechocystis* producing heterologous 2-oxoglutarate:ferredoxin oxidoreductase (CtOGOR). The photoautotrophic growth of WT-ogor (circle) and WT-1211 (triangle) were recorded in the presence (closed symbols) and absence (open symbols) of 1 mM IPTG (**a**). Specific growth rates were determined at exponential phases. Transcript levels of *korB* (**b**) at exponential phase was determined by RT-qPCR using 16S rRNA as a reference gene. The cellular activity of CtOGOR (**c**) was determined and normalized by protein level (mg). Significances were assessed by Student’s *t*-test: ns, *p* > 0.05; *, *p* ≤ 0.05; **, *p* ≤ 0.01; ***, *p* ≤ 0.001 (*n* = 3).

Next, we attempted to enhance the carbon fixation of *Synechocystis* by introducing CtOGOR (Figure 4). CtOGOR was heterologously produced in wild type (WT) *Synechocystis* by expressing pSL-ogor (the resulting recombinant strain called WT-ogor), in which the CtOGOR gene was expressed with the IPTG (1 mM)-inducible *trc* promoter. The photoautotrophic growth of WT-ogor was faster than that of the control strain of *Synechocystis* WT, which carried the empty pSL1211 vector (the resulting recombinant strain called WT-1211), especially during the exponential phase in the presence of IPTG (Figure 5a). The growth of control strain WT-1211 was 3.06 ± 0.02 × 10^−2^ h^−1^, which was comparable with the growth rate of 2.75 × 10^−2^ h^−1^ determined with *Synechocystis* WT cell in other study [37]. The specific growth rate of WT-ogor (+IPTG) was approximately 36% higher than that of WT-1211 (+IPTG). However, the OD at the stationary phase was identical in all strains examined. The pH of the culture medium BG-11 was initially 7.5; however, it reached 9.0 during the stationary phase (Figure S2a,c). We suspected that an alkaline pH may decrease the effect of CtOGOR, thus leading to the same biomass in the stationary phase. Therefore, the cell culture was titrated to pH 7.5 every 12 h to maintain a constant culture pH. However, there was no difference in growth or biomass between WT-ogor (+IPTG) and WT-1211 (+IPTG) (Figure S2b,d).

To confirm the production of CtOGOR in *Synechocystis*, cells were harvested during the exponential phase and the gene expression of CtOGOR was examined by RT-qPCR. The expression level of *korB* in WT-ogor (+IPTG) was 15-fold higher than that in WT-ogor (−IPTG), indicating the induced expression of *korAB* by IPTG (Figure 5b). This result was confirmed again by the determination of cellular OROR activity, in which only WT-ogor (+IPTG) had significant cellular OGOR activity (Figure 5c). Although the promoter of pSL1211 is tightly regulated by IPTG, there was some leaky expression even in the absence of IPTG, which was observed with WT-ogor (−IPTG), leading to a slight increase in the specific growth rate (Figure 5a) and transcript level (Figure 5b) compared with the control values of WT-1211. However, no difference was detected in the spectral analysis of the membrane fractions (Figure S3), which implies no apparent effect of CtOGOR production on PS complexes. Since the growth of *Synechocystis* is sensitive to the intrinsic RuBisCO level [16], cellular RuBisCO was also examined. The expression levels of *rbcL* (i.e., the gene encoding the large subunit of RuBisCO) (Figure S4a) and cellular RuBisCO activities (Figure S4b) were the same in both WT-ogor and WT-1211.

**Figure 6:**
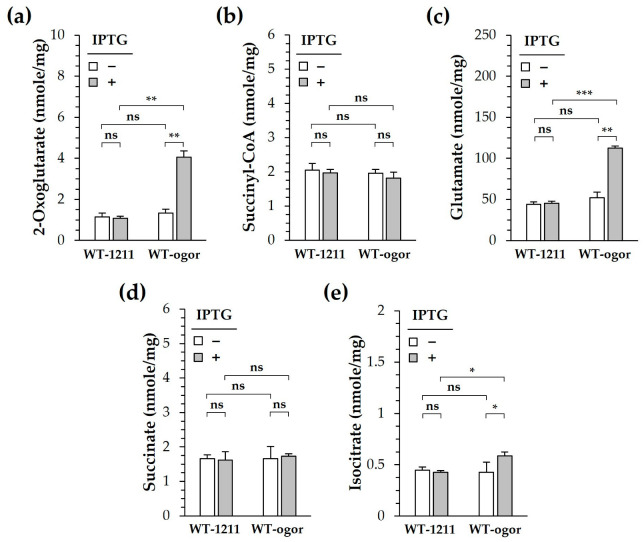
Cellular metabolite levels of *Synechocystis* producing 2-oxoglutarate:ferredoxin oxidoreductase (CtOGOR). WT-ogor and WT-1211 were grown photoautotrophically in the presence and absence of 1 mM IPTG. Cells were harvested at the exponential phase for metabolite analysis. The cellular level of 2-oxoglutarate (**a**), succinyl-CoA (**b**), glutamate (**c**), succinate (**d**) and isocitrate (**e**) were determined and normalized by total protein contents in the cells (mg). Significances were assessed by Student’s *t*-test: ns, *p* > 0.05; *, *p* ≤ 0.05; **, *p* ≤ 0.01; ***, *p* ≤ 0.001 (*n* = 3).

Because CtOGOR connects the metabolic flow between succinyl-CoA and 2-oxoglutarate, the cellular levels of 2-oxoglutarate were determined using the deprotenized lysate obtained from cells grown exponentially under photoautotrophic conditions. 2-Oxoglutarate—the carboxylation product of CtOGOR—increased 4-fold in WT-ogor (+IPTG) compared to that in WT-ogor (-IPTG) and WT-1211 controls (Figure 6a). Meanwhile the cellular level of succinyl-CoA—a decarboxylation product of CtOGOR—did not change. Likewise, no change in succinate levels was observed (Figure 6d). These results reinforce the notion that CtOGOR is preferred for carboxylation over decarboxylation. Isocitrate level of WT-ogor (+IPTG) increased by approximately 30% compared to that of the controls (Figure 6e), which may be attributed to the carboxylation reaction of Icd. The reversibility of Icd was demonstrated by the kinetic analysis (Table S1). Alternatively, Icd may be inhibited by an increased level of 2-oxoglutarate [40], resulting in the accumulation of isocitrate in cells. Since 2-oxoglutarate is the substrate of GOGAT and GDH, glutamate levels increased by approximately 2.5-fold in WT-ogor (+IPTG) compared to those in WT-ogor (-IPTG) and WT-1211 controls (Figure 6c). Moreover, glutamate is known to significantly affect the biomass yield of *Synechocystis* [18].

## 4. Discussion

Several studies have characterized OGOR from diverse organisms such as *Halobacterium halobium* [3], *Thermococcus litoralis* [4], *Sulfolobus* sp. Strain 7 [5], *Thauera aromatica* [6], *Sulfolobus tokodaii* [7], and *Magnetococcus marinus* [8]. Although, oligomeric states vary depending on the origin of the enzyme, OGOR is composed of two subunits in the states of (αβ)_1_ or (αβ)_2_. Additionally, OGORs have 1–2 mole of (4Fe-4S) and 1–2 mole of TPP per enzyme in most cases. CtOGOR is composed of the (αβ)_2_ form and has two (4Fe-4S) clusters and one TPP, sharing features with previously reported OGOR enzymes.

CtOGOR requires Fd as a mediator for electron transfer. Although there may be a partner Fd for CtOGOR in *C. tepidum*, which is yet to be found, the major concern of this study was whether CtOGOR could work with *Synechocystis* Fds in cells. *Synechocystis* possesses nine Fds named Fed1–9 [41], the reduction potentials of which are in the range of −240 to −680 mV [42]. To examine the in vitro reaction of CtOGOR, we selected Fed2 (slr1382) as a representative, which has a [2Fe-2S] cluster and the highest reduction potential (−243 mV) [43]. *M. marinus* (Mm) OGOR was assumed to have a reduction potential of −545 mV and it exhibited in vitro carboxylation reaction with three Fds—MmFd1 (−635 mV and −485 mV for each of two 4Fe-4S clusters), MmFd2 (−520 mV for each of two 4Fe-4S clusters), and MmFd3 (−380 mV and −233 mV for each of two 4Fe-4S clusters) [8]. Although MmFd1 with the lowest negative reduction potential showed the highest carboxylation activity, the other Fds with higher potentials also showed comparable activities [8]. Thus, the in vitro carboxylation of CtOGOR by *Synechocystis* Fed2 can be explained. The in vivo partner Fd of *Synechocystis* suitable for carboxylation and decarboxylation by CtOGOR remains to be determined.

*Synechocystis* performs oxygenic photosynthesis generating O_2_ through water splitting, which could affect CtOGOR activity. O_2_ is not a crucial issue because the intracellular O_2_ concentration is extremely low in *Synechocystis* cells (0.064 μM; ~0.0001% partial pressure) [44]. Although the low O_2_ could still affect CtOGOR activity in cells, the steady-state enzyme level in *Synechocystis* was thought to be constitutively maintained due to the overexpression by plasmid.

Carbon fixation is a rate-limiting step in the cell growth and productivity (e.g., biomass) of *Synechocystis* [15]. We demonstrated that the overexpression of RuBisCO [16], Fructose-1,6-/sedoheptulose 1,7-biphosphatase [17], and fructose-bisphosphate aldolase [17], which are enzymes involved in the CBB cycle, could enhance the growth of *Synechocystis* (under high light-intensity). Additionaly, several carboxylases positively affect photoautotrophic growth and biomass formation in *Synechocystis*. Overexpression of phosphoenolpyruvate carboxylase increases the growth rate of *Synechocystis* by 43% [45] and ethylene productivity by 64% (under low light-intensity) [46]. Overexpression of acetyl-CoA carboxylase originating from *E. coli* enhanced lipid production yield in *Synechocystis* by 3.6-fold [47]. Although the detailed mechanisms vary from case to case, they commonly imply that the increased growth and productivity of *Synechocystis* could be achieved by promoting the integration of CO_2_ (or HCO_3_^−^) into the cellular metabolite pools.

The reaction of heterologously produced CtOGOR seems to be inclined toward carboxylation in *Synechocystis*. This was demonstrated by the result that CtOGOR production increased 2-oxoglutarate levels (Figure 6a) but did not alter succinyl-CoA levels. At least five enzymes (pathways) were involved in 2-oxoglutarate metabolism: Icd, GOGAT, GdhA, OgdA, and CtOGOR (decarboxylation reaction) (Figure 4). The *K*_m_ values (mM) for 2-oxoglutarate were as follows: Icd, 1.003 (Table S1); GOGAT, 0.04 [48]; GdhA, 1.8 [49]; OgdA, 21 [50]; and CtOGOR, 1.689 (Table 2). The cellular 2-oxoglutarate level was estimated to be 0.02–0.20 mM [40], which is far below the *K*_m_ values of the enzymes. Thus, the amount of 2-oxoglutarate the enzymes take in their reaction would be determined primarily by *K*_m_ of the enzymes. Compared with the other enzymes, *K*_m_ of GOGAT is considerably lower, which means that GOGAT has the highest affinity for 2-oxoglutarate. Accordingly, 2-oxoglutarate formed by CtOGOR would be mainly metabolized by GOGAT to glutamate.

The 2-oxoglutarate reportedly acts as a signaling molecule for carbon-nitrogen balance [51] mainly via the PII signal transduction protein [52]. Indeed, the C/N ratio of cyanobacteria is maintained at 5:1 [53]. However, the BG-11 [19] minimal medium used in this study contained a sufficient amount of a fixed nitrogen source (NO_3_^−^) and no fixed carbon source, which could result in a low C/N ratio in *Synechocystis*. Under these conditions, exogenous CtOGOR could contribute to elevating the fixed carbon level via carboxylation, leading to an appropriate C/N ratio for optimal growth.

Glutamate generated by CtOGOR can be utilized to produce building blocks of cellular components [54]. As an amino acid, glutamate itself participates in de novo protein synthesis (translation) and is used for the synthesis of other amino acids such as glutamine, proline, and arginine. More than 50% of cell components in *Synechocystis* are proteins [38] and glutamate has a more positive effect on the enhancement of biomass yield compared with other metabolites of the CBB cycle and central carbon metabolism [18]. *Synechocystis* adopts the C5 pathway to synthesize 5-aminolevulinic acid and glutamate is the main starting molecule of crucial biosynthetic pathways such as chlorophyll *a*, heme, phycocyanobilin, phycoerythrobilin and cobalamin [39]. Furthermore, glutamate is used to synthesize glutathione, a well-known cellular antioxidant [55]. Thus, an increase in cellular glutamate levels would have positive effects on cell growth under carbon-limiting photoautotrophic conditions.

## 5. Conclusions

This study characterized a CO_2_-fixing enzyme CtOGOR of OFOR family and found it similarly forms heterotetramer (αβ)_2_ and contains two (4Fe-4S) clusters and one TPP per (αβ)_1_ protomer. Exogenous production of CtOGOR in *Synechocystis* resulted in the elevation of the levels of 2-oxoglutarate, a central metabolite linking carbon and nitrogen metabolism. Glutamate level increased accordingly, and the photoautotrophic growth rate increased. However, carboxylation by CtOGOR may not largely affect carbon availability in the cell. Thus, further study should focus on examining the effect of CtOGOR production during higher carbon availability via over-producing CBB cycle enzymes including RuBisCO of cyanobacteria under photoautotrophic conditions.

## Figures and Tables

**Table 1:** Cofactor contents of 2-oxoglutarate:ferredoxin oxidoreductase (CtOGOR).

Content	Number per αβ-Protomer *
Fe	7.51 ± 0.51
S **	7.65 ± 0.12
TPP	0.77 ± 0.07

* Determined after reconstitution of the (Fe-S) cluster and thiamine pyrophosphate (TPP). ** Total sulfur from sulfide plus sulfane.

**Table 2:** Enzyme kinetic parameters of 2-oxoglutarate:ferredoxin oxidoreductase (CtOGOR).

Direction of Reaction	Substrate	*K*_m_ (mM)	*k*_cat_ (s^−1^) *	*k*_cat_/*K*_m_ (s^−1^ mM^−1^)
Carboxylation	Succinyl-CoA	0.024 ± 0.002	2.6 ± 0.1	110.2 ± 3.5
	CO_2_ **	0.468 ± 0.045	2.7 ± 0.1	6.5 ± 0.2
Decarboxylation	2-Oxoglutarate	1.689 ± 0.092	8.4 ± 0.1	5.0 ± 0.1
	CoA	0.042 ± 0.015	8.1 ± 0.2	191.7 ± 2.4

* Turnover number per αβ-protomer. ** Concentration was estimated by that of sodium bicarbonate.

## Data Availability

Not applicable.

## Supplementary Materials

The following supporting information can be downloaded at: https://www.mdpi.com/article/10.3390/biology12010059/s1. Figure S1: Uncropped SDS-polyacrylamide gel and Western blots of CtOGOR; Figure S2: Effect of pH titration to 7.5 on the photoautotrophic growth of *Synechocystis* WT producing CtOGOR; Figure S3: Membrane spectra of *Synechocystis* WT producing CtOGOR; Figure S4: Transcript level and cellular activity of RuBisCO in *Synechocystis* WT producing CtOGOR; Table S1: Enzyme kinetic parameters of isocitrate dehydrogenase of *Synechocystis*.
