# Supplementary material for: Photoautotrophic Growth Rate Enhancement of Synechocystis sp. PCC6803 by Heterologous Production of 2-Oxoglutarate:Ferredoxin Oxidoreductase from Chlorobaculum tepidum
Source: Biology (Basel). 2022 Dec 29;12(1):59. doi: 10.3390/biology12010059 (PMC9855186; doi:10.3390/biology12010059)
Supplement: Supplementary file 1 [file biology-12-00059-s001.zip › biology-2099740-supplementary.pdf]

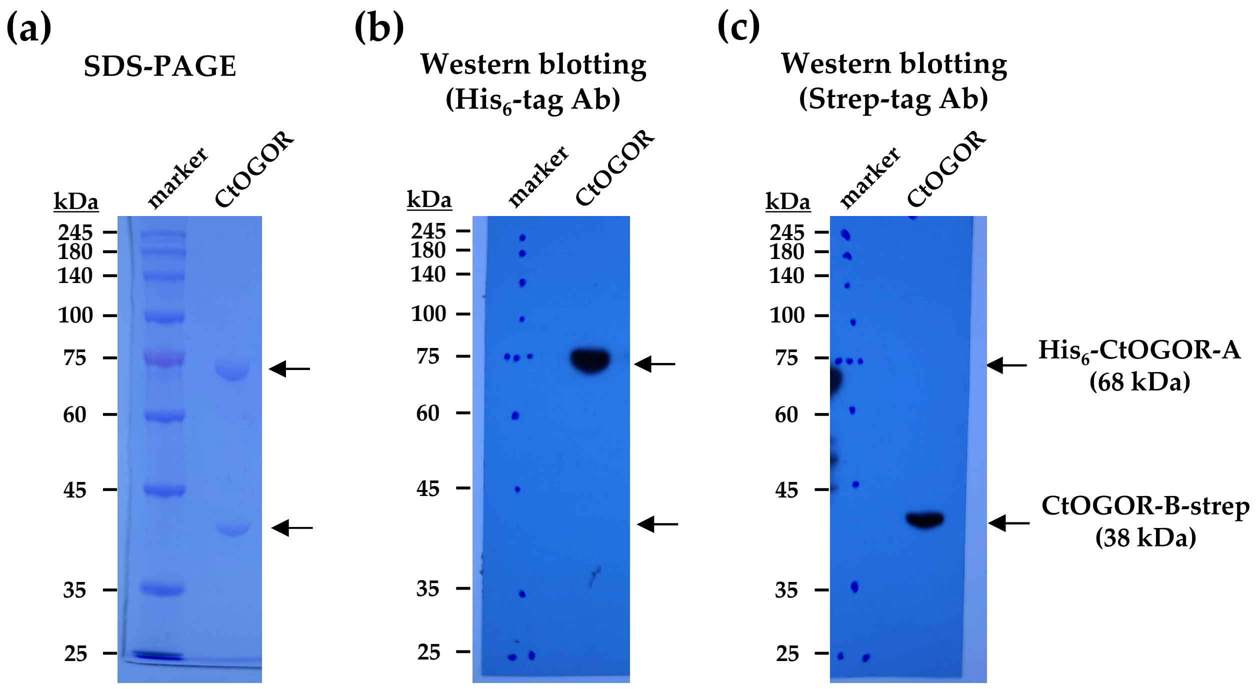

Figure S1. Uncropped SDS-polyacrylamide gel and western blots of CtOGOR.
SDS-polyacrylamide gel of the purified CtOGOR (a) and the western blot films with His$_6$-tag Ab (b) and Strep-tag Ab (c) were shown. SDS-polyacrylamide gel a, blot b, and blot c correspond to Figure 2b, Figure 2c, and Figure 2d, respectively.

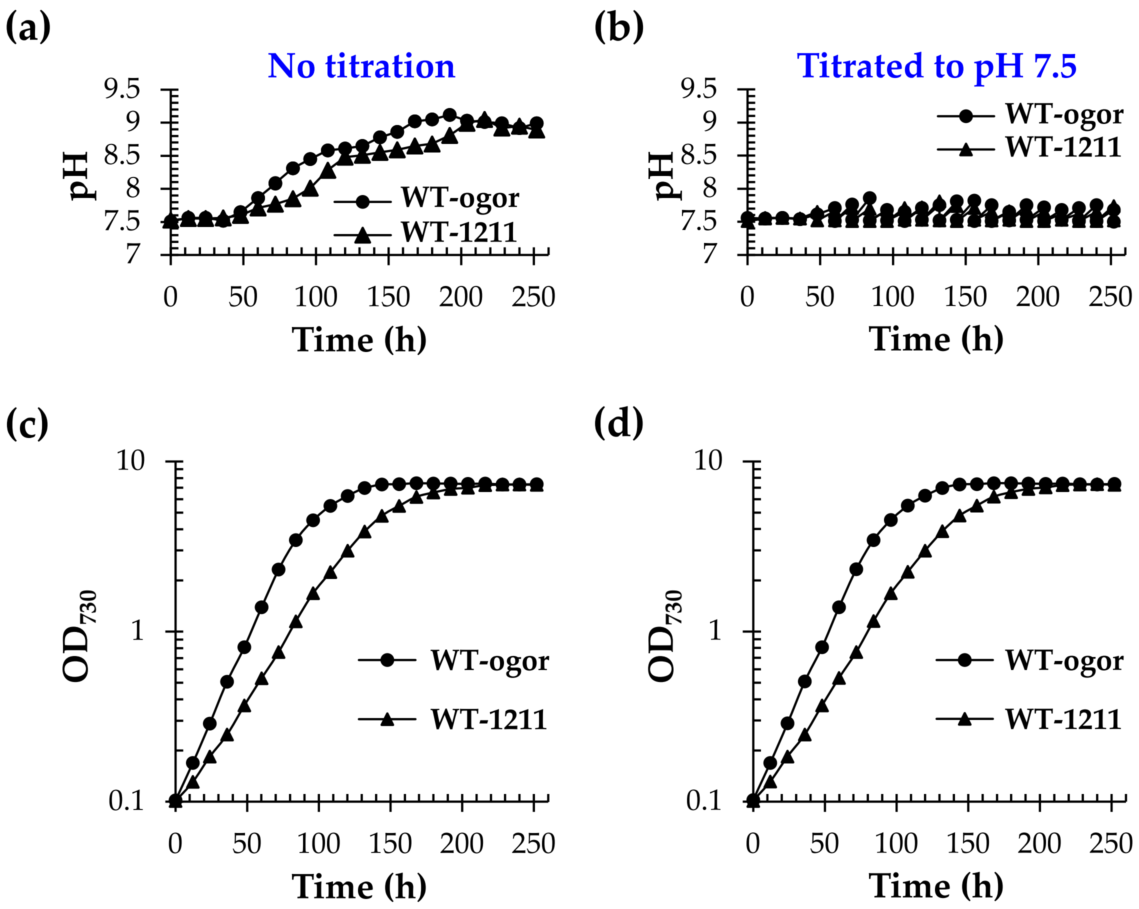

Figure S2. Effect of pH titration to 7.5 on the photoautotrophic growth of *Synechocystis* WT producing CtOGOR.

WT-ogor and WT-1211 were grown photoautotrophically in the presence of 1 mM IPTG, while the pH (a and b) and growth (c and d) were monitored. One group of cells was not titrated (a and c), but the other group was titrated to pH 7.5 every 12 h with HCl and NaOH (b and d).

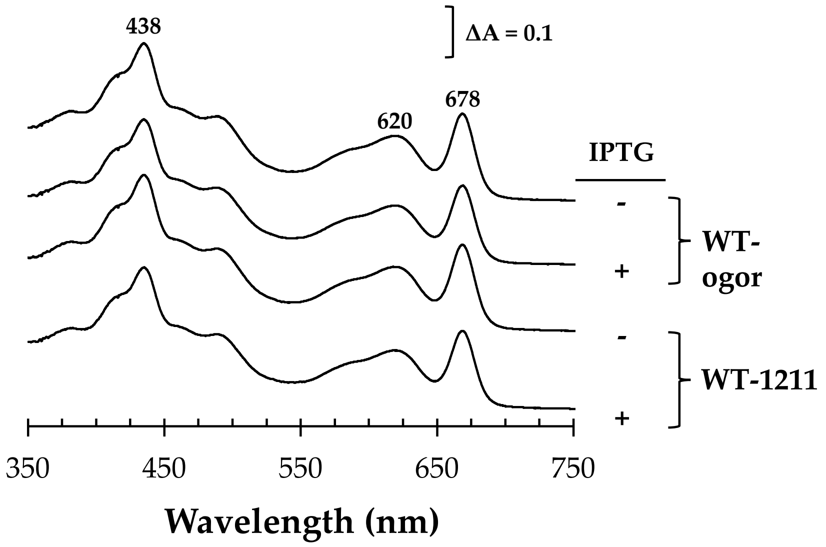

Figure S3. Membrane spectra of *Synechocystis* WT producing CtOGOR.
WT-1211 and WT-ogor were cultured photoautotrophically in the presence or absence of 1 mM IPTG, which were harvested at exponential phase. Cells were lysed and crude membranes were isolated by centrifugation. Samples were normalized by total protein level. The λ_{max} was illustrated over major peaks.

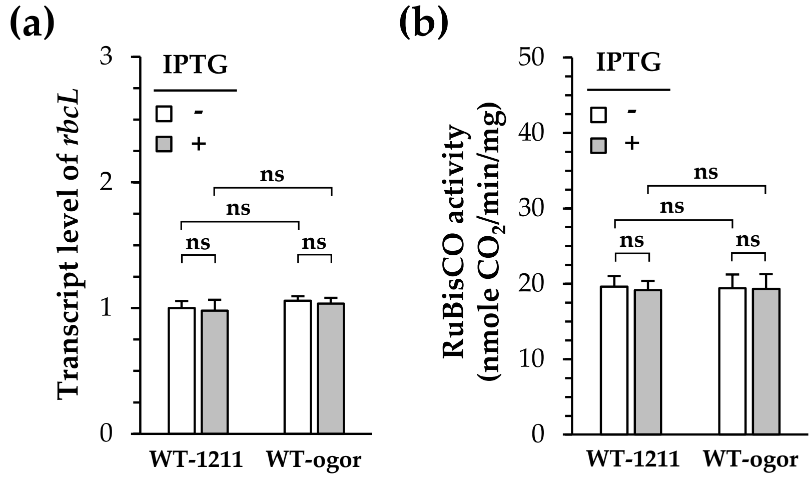

Figure S4. Transcript level and cellular activity of RuBisCO in *Synechocystis* WT producing CtOGOR. WT-1211 and WT-ogor cultured photoautotrophically in the presence or absence of 1 mM IPTG were harvested at exponential phase. Transcript levels of gene for large subunit of RuBisCO (*rbcL*) were subject to RT-qPCR with 16S rRNA gene as a reference gene (a). Cellular RuBisCO activities were determined and normalized by protein level (mg) (b). Significances were assessed by Student's *t*-test: ns, $p > 0.05$; *, $p \leq 0.05$; **, $p \leq 0.01$; ***, $p \leq 0.001$ ($N = 3$).

Table S1. Enzyme kinetic parameters of isocitrate dehydrogenase of *Synechocystis*.

Direction of reaction	Substrate	K_m (mM)	k_{cat} (s^{-1})	k_{cat}/K_m (s^{-1} mM^{-1})
**Carboxylation**	**2-Oxoglutarate**	1.003 ± 0.092	12,647±633	12,254 ± 633
	**CO_2***	0.423 ± 0.088	11,862±1,260	28,952 ± 1,507
**Decarboxylation**	**Isocitrate**	0.121 ± 0.013	90,196 ± 1,812	745,421 ± 14,975

* Concentration was estimated from that of sodium bicarbonate.
